# Multiple Genome Sequences of *Helicobacter pylori* Strains of Diverse Disease and Antibiotic Resistance Backgrounds from Malaysia

**DOI:** 10.1128/genomeA.00687-13

**Published:** 2013-09-19

**Authors:** Vellaya Rehvathy, Mun Hua Tan, Selva Perumal Gunaletchumy, Xinsheng Teh, Susana Wang, Primo Baybayan, Siddarth Singh, Meredith Ashby, Nadeem O. Kaakoush, Hazel M. Mitchell, Laurence J. Croft, Khean Lee Goh, Mun Fai Loke, Jamuna Vadivelu

**Affiliations:** Department of Medical Microbiology, University of Malaya, Kuala Lumpur, Malaysiaa; Malaysian Genomics Resource Centre Berhad, Kuala Lumpur, Malaysiab; Pacific Biosciences, Menlo Park, California, USAc; PacBio Singapore, Teletech Park, Singapored; School of Biotechnology and Biomolecular Sciences, University of New South Wales, Sydney, Australiae; Department of Medicine, University of Malaya, Kuala Lumpur, Malaysiaf

## Abstract

*Helicobacter pylori* causes human gastroduodenal diseases, including chronic gastritis and peptic ulcer disease. It is also a major microbial risk factor for the development of gastric adenocarcinoma and mucosa-associated lymphoid tissue (MALT) lymphoma. Twenty-one strains with different ethnicity, disease, and antimicrobial susceptibility backgrounds were sequenced by use of Illumina HiSeq and PacBio RS platforms.

## GENOME ANNOUNCEMENT

Clinical outcomes induced by *Helicobacter pylori* vary with individuals (1), especially among those with different ethnic origins (2). In fact, this is a hallmark of the Gram-negative curved bacterium that resides in the human stomach. Hypothetically, this variation could be due to the diversity of pathogenic genes present in the *H. pylori* strains infecting different ethnic groups (1), the geographically distinct DNA polymorphisms of *H. pylori* (2, 3), or the lifestyles of people of different ethnic groups (4). This phenomenon is not exceptional in multiracial Malaysia (5). Together with 10 previously announced genome sequences (6), which have been reassembled using newer algorithms, we present here 21 genome sequences of *H. pylori* strains isolated from patients with different ethnicities, disease statuses, and antimicrobial susceptibility patterns who were attending the endoscopy unit at the University of Malaya Medical Center (UMMC) (Table 1).

**Table 1:** Sequencing statistics, genome information, strain characteristics, and accession numbers for 21 *H. pylori* strains^*c*^

Strain^*c*^	No. of contigs (≥200 bp)	ABySS *k*-mer	No. of scaffolds	No. of bases	Maximum scaffold size (bp)	Mean scaffold size (bp)	Median scaffold size (bp)	*N*_50_ (bp)	*N*_90_ (bp)	GC content (%)	No. of predicted genes (≥50 amino acids)	No. of annotated genes (E value <10^–10^, PID ≥80%)	Remarks	Accession no.
FD662	67	47	24	1,660,698	406,404	69,196	35,327	212,690	31,444	38.92	1,623	1,556	Malay; NUD	AKHT00000000^*a*^
FD703	89	51	27	1,678,101	311,702	62,152	22,813	162,651	40,720	39.01	1,603	1,543	Malay; NUD	AKHS00000000^*a*^
FD719	79	53	24	1,643,472	313,579	68,478	51,079	97,163	37,907	39.07	1,609	1,543	Malay; NUD	AKHU00000000^*a*^
UM084	34	73	21	1,657,009	332,847	78,905	51,819	261,002	39,185	39.05	1,576	1,516	Malay; PUD; MZ	AUSO00000000^*a*^
FD423	115	51	26	1,625,381	316,018	62,515	45,159	97,115	37,787	39.09	1,599	1,538	Indian; NUD	AKHM00000000^*a*^
FD430	130	57	30	1,643,626	212,462	54,788	41,777	111,117	36,409	39.03	1,626	1,567	Indian; NUD	AKHN00000000^*a*^
FD535	81	51	24	1,673,398	399,528	69,725	39,365	117,068	38,267	39.04	1,608	1,554	Indian; NUD	AKHP00000000^*a*^
UM067	44	63	23	1,681,714	564,610	73,118	35,626	102,467	33,274	39.02	1,610	1,547	Indian; PUD; MZ	AUSN00000000^*a*^
UM114	36	49	24	1,709,511	345,511	71,230	36,863	259,963	31,894	38.91	1,623	1,559	Indian; PUD; MZ	AUSS00000000^*a*^
UM037	60	67	39	1,724,611	234,132	44,221	29,236	80,609	26,936	38.89	1,645	1,574	Indian; stomach fundus tumor; CH	AUSI00000000,^*a*^ CP005492^*b*^
FD506	105	51	26	1,618,298	339,042	62,242	33,881	135,883	33,881	38.66	1,578	1,533	Chinese; NUD	AKHO00000000^*a*^
FD568	114	59	24	1,613,149	385,714	67,215	31,934	169,920	38,934	38.64	1,566	1,528	Chinese; NUD	AKHQ00000000^*a*^
FD577	74	57	26	1,627,035	362,981	62,578	41,783	125,077	38,935	38.64	1,590	1,554	Chinese; NUD	AKHR00000000^a^
UM065	39	63	24	1,587,249	334,064	66,135	40,061	163,534	32,497	38.90	1,500	1,461	Chinese; PUD	AUSM00000000^*a*^
UM066	34	65	24	1,694,309	319,894	70,596	39,327	146,858	35,777	38.64	1,590	1,562	Chinese; PUD	AUSJ00000000,^*a*^ CP005493^*b*^
GC26	111	55	27	1,626,266	328,361	60,232	31,457	152,049	31,457	38.64	1,592	1,561	Chinese; GC	AKHV00000000^*a*^
UM023	35	63	15	1,624,154	485,260	108,277	53,518	183,178	39,954	38.74	1,562	1,518	Chinese; PUD; MZ	AUSK00000000^*a*^
UM077	53	65	28	1,620,877	328,671	57,888	35,423	187,040	35,423	38.78	1,565	1,527	Chinese; PUD; FQ	AUSQ00000000^*a*^
UM038	45	63	27	1,762,854	411,489	65,291	38,877	94,812	35,087	38.42	1,663	1,602	Chinese; NUD; CH, FQ	AUSL00000000^*a*^
UM085	50	75	29	1,645,640	341,517	56,746	39,737	94,888	39,574	38.72	1,568	1,524	Chinese; NUD; CH, FQ	AUSP00000000^*a*^
UM111	38	59	29	1,663,383	245,602	57,358	32,048	110,134	30,450	38.68	1,581	1,536	Chinese; NUD; CH, MZ	AUSR00000000^*a*^

a Illumina HiSeq 2000 (draft whole-genome sequence).

b PacBio SMRT (complete genome sequence).

c PID, percent identity; CH, clarithromycin resistant; FQ, fluoroquinolone resistant; GC, gastric cancer; MZ, metronidazole resistant; NUD, nonulcer dyspepsia; PUD, peptic ulcer disease.

*H. pylori* DNA was isolated using the RTP bacteria DNA minikit (Invitek GmbH, Berlin, Germany). Whole-genome sequencing was performed using 100-base, paired-end reads on the Illumina HiSeq 2000 instrument (Illumina, Inc., San Diego, CA) at the Malaysian Genomics Resource Centre Berhad (MGRC) (Kuala Lumpur, Malaysia). Assemblies were performed for each sample at optimal *k*-mers using the ABySS assembler version 1.3.4 (7). Assembled contigs were scaffolded with SSPACE using paired-end read information from each sample (8). Gene sequences were predicted from assembled scaffolds using GeneMark version 2.5 (9). Predicted gene sequences were aligned against the UniProt (Swiss-Prot/TrEMBL) database using SynaSearch (MGRC) for annotation.

Two strains, UM037 and UM066, were also sequenced using Pacific Biosciences RS sequencing technology (Pacific Biosciences, Menlo Park, CA), yielding >20× average genome coverage. Each sample was prepared as a 10-kb insert library using C2 chemistry and sequenced on 8 single-molecule real-time (SMRT) cells. *De novo* assembly of the read sequences was carried out using continuous long reads (CLR) following the Hierarchical Genome Assembly Process (HGAP) workflow (PacBio DevNet; Pacific Biosciences) as available in SMRT Analysis v2.0. The genomes were annotated with the NCBI (National Center for Biotechnology Information) Prokaryotic Genomes Automatic Annotation Pipeline. Using the PacBio workflow, the *H. pylori* UM037 and UM066 genome sequences were assembled as single contigs of 1,692,823 bp and 1,660,425 bp, respectively. The NCBI annotation predicted 1,677 and 1,637 open reading frames (ORFs) (1,637 and 1,595 annotated genes) for UM037 and UM066, respectively.

The availability of these *H. pylori* genome sequences from individuals from different ethnic backgrounds with distinct clinical presentations provides the research community with a resource for detailed investigations into the genetic elements that correlate with bacterial evolution, compensatory mechanisms, host adaptation, gastric pathogenesis, and selective pressure exerted by antimicrobial agents. Furthermore, these sequencing data sets also enable the comparison of Illumina HiSeq and PacBio RS sequencing platforms for *H. pylori* genomes.

### Nucleotide sequence accession numbers.

The *H. pylori* genome sequences described in this paper have been deposited as draft whole-genome shotgun projects in DDBJ/EMBL/GenBank under the accession numbers stated in Table 1. The versions described in this paper are the first versions.
